# The Development and Content of the Vocational Advice Intervention and Training Package for the Study of Work and Pain (SWAP) Trial (ISRCTN 52269669)

**DOI:** 10.1007/s10926-018-9799-1

**Published:** 2018-07-07

**Authors:** G. Sowden, C. J. Main, D. A. van der Windt, K. Burton, G. Wynne-Jones

**Affiliations:** 1 Arthritis Research UK Primary Care Centre, Research Institute for Primary Care and Health Sciences, Keele University, Keele, Staffordshire ST5 5BG UK; 2 Interface Multidisciplinary Pain Assessment and Community Treatment Service Haywood Hospital, High Lane, Burslem, Stoke-On-Trent, ST6 7AG UK; 3 Centre for Applied Research in Health, University of Huddersfield, Huddersfield, UK

**Keywords:** Musculoskeletal, Work, Case management

## Abstract

*Purpose* There are substantial costs associated with sickness absence and struggling at work however existing services in the UK are largely restricted to those absent from work for greater than 6 months. This paper details the development of an early Vocational Advice Intervention (VAI) for adult primary care consulters who were struggling at work or absent due to musculoskeletal pain, and the structure and content of the training and mentoring package developed to equip the Vocational Advisors (VAs) to deliver the VAI, as part of the Study of Work and Pain (SWAP) cluster randomised trial. *Methods* In order to develop the intervention, we conducted a best-evidence literature review, summarised evidence from developmental studies and consulted with stakeholders. *Results* A novel early access, brief VAI was developed consisting of case management and stepped care (three steps), using the Psychosocial Flags Framework to identify and overcome obstacles associated with the health-work interface. Four healthcare practitioners were recruited to deliver the VAI; three physiotherapists and one nurse (all vocational advice was actually delivered by the three physiotherapists). They received training in the VA role during a 4-day course, with a refresher day 3 months later, along with monthly group mentoring sessions. *Conclusions* The process of development was sufficient to develop the VAI and associated training package. The evidence underpinning the VAI was drawn from an international perspective and key components of the VAI have the potential to be applied to other settings or countries, although this has yet to be tested.

## Background

Across Europe sickness absence from work costs approximately 2.5% of Gross Domestic Product [1] and in Britain it costs the economy an estimated £15 billion/year, including lost productivity or output, time spent on sickness absence management and healthcare [2]. A major cause of sickness absence is musculoskeletal pain [3], and sickness absence in those with musculoskeletal pain is associated with increased physical and mental health morbidity [4, 5] which in turn, can affect levels of function, quality of life, family and other relationships [6, 7]. Individual and societal costs are likely to increase as populations grow and age [1]. It is widely recognised that the longer an employee is off work, the lower their chances of returning to work (RTW) and the greater their risk of job loss [8, 9]. Consequently, there is consensus that early intervention with good vocational advice can prevent longer-term absence and disability in those with musculoskeletal disorders [10]. Early intervention can also reduce the length of sickness absence by facilitating early return to work, and improve the chances of sustained retention in work [10, 11] thus providing a cost-benefit for employers, health purchasers and providers of wage replacement benefits [10]. Models have been developed to underpin the delivery of high intensity, multidisciplinary vocational rehabilitation, and descriptions of these interventions have been summarised in systematic reviews [12]. There is also evidence summarising the specific components necessary for vocational rehabilitation in those who have been away from the workplace for a longer period [10]. However, many of these studies either deliver vocational advice within the workplace, are focussed on those with longer term absence or describe complex multidisciplinary interventions which are difficult to implement in clinical practice outside the clinical trial environment [13]. Although early intervention is advocated to prevent employees transitioning from short-term to longer-term absence [10], it is not clear exactly what an early intervention should include, or what models should underpin its delivery. The delivery seems particularly problematic: it is known that adoption rates for complex interventions in primary care are low [14], so it is important to take appropriate steps to ensure fidelity between the evidence and what is actually delivered. The Study of Work and Pain (SWAP) trial was delivered in six general practices in the United Kingdom and aimed to determine whether the addition of an early vocational advice service to best current primary care could reduce work absence in patients consulting their GP for musculoskeletal pain who were either absent from work or struggling to remain in work because of their pain [15–17]. The trial demonstrated that the Vocational Advice Intervention (VAI) significantly reduced days absent over 4 months and was cost effective compared to best care [16]. This paper details the development of the early VAI, and describes the structure and content of the training and mentoring package developed to equip the Vocational Advisors (VAs) to deliver it. It is a companion paper to three other papers, the SWAP trial protocol paper [15], the SWAP trial results paper [16] and a qualitative paper exploring the acceptability of the VAI to general practitioners, the VAs and patients [17].

## Methods

The development of the VAI and the associated training and support package to equip the VAs to deliver it was consistent with the new Medical Research Council guidance on developing and evaluating complex interventions [18]. Existing evidence and theory was identified by conducting a review of current literature and policy documents and new evidence was developed through developmental studies. In addition, stakeholders were consulted.

### Best-Evidence Review of Literature and Policy Documents

A best-evidence review of current research evidence, policy documents and grey literature was conducted in order to identify best practice, and methods that have been used successfully to promote working with health conditions [e.g., 10, 19–22]. These publications were collated and discussions between the team then identified the salient points, which were then taken into account in designing the VAI. These were that: (1) the VAI needed to be delivered early; (2) patients should be accessed easily, for example, via the GP as many people off sick and struggling at work consult with a GP; (3) the VAs needed to work collaboratively with patients to identify and provide practical solution to help them to remain or return to work; (4) the VAI should involve all stakeholders, although this is particularly challenging when intervening early. The structure, format, content and methods of delivery of the VAI training were informed by evaluations of training offered to healthcare professionals (HCPs) [17] as part of two previous trials, STarT Back [23] and IMPaCT Back [24]. Additional sources included; (i) peer reviewed publications and evidence based guidelines [e.g., 17, 22, 25–27]; (ii) existing training in vocational case management, occupational health and vocational rehabilitation (e.g. GS has contributed to the development and delivery of Salford University Masters modules in the UK); (iii) relevant codes of practice and standards [e.g., 28]; (iv) competencies [e.g., 21]; and (v) recommendations for training [e.g., 29].

### Evidence from Developmental Studies

Members of the research team conducted a number of developmental studies which informed the design of the intervention. The first study, investigating trends in GP sickness certification over time helped identify the need for a VAI located in primary care [30]. A qualitative interview study among HCPs showed that physiotherapists were more likely to ask patients about work than GPs, that there was a tension between GPs’ perceived roles as patient advocate and gatekeeper to sickness benefits, and that both GPs and physiotherapists felt frustrated about the limited control they had over the patient’s workplace [31]. The third study used data from a cohort study to assess the outcomes of sickness certification for patients presenting with back pain in general practice. This indicated that receiving a sick not does not necessarily benefit patients as clinical outcomes were similar between those receiving and those not receiving a sickness certificate (aside from anxiety which was higher in those receiving a sickness certification) and sickness certification was associated with increased costs, mainly due to productivity losses [32]. Finally, a prospective cohort study among 505 employees (public sector organisations in the UK) identified reasons why people might struggle at work. The results demonstrated that whilst health variables accounted for the largest proportion of explained variance, work characteristics and conditions of employment, as well as an individual’s perceptions of work were also risk factors for absence and reduced performance in the workplace [33]. This work highlighted the need for the VAI and guided the decision to locate the VAI in primary care, staffed with non-medical health professionals.

### Consultation with Health Professionals and Patients

In order to guide the development of the VAI, a number of consultations were held between 2010 and 2012, to gain the views of representatives from key stakeholder groups. These consultations were held at Keele University, participants were provided with an overview of the research question and proposed methods of the SWAP trial. Facilitators then presented the topics for discussion and supported these discussions through summarising key points and feeding back to the group to ensure that views were accurately represented. Meetings took place with six local GPs and a multidisciplinary Clinical Advisory Group (CAG) including physiotherapists, GPs, rheumatologists and a vocational case manager. Stakeholders were part of already established clinical advisory groups affiliated with the Arthritis Research UK Primary Care Centre and members were invited by letter to participate. The purpose of these meetings was to provide clinical opinion, specifically on aspects of the intervention development. In addition, two Patient and Public Involvement and Engagement (PPIE) meetings were held in 2010 and 2012, with individuals who had experience of living with musculoskeletal pain. Both meetings allowed PPIE members to discuss the design of the SWAP trial, and the potential implications for patients taking part, with suggestions for amendments to maximise participation and also consideration of the content of the VAI and whether it was likely to address the vocational advice needs of those working with musculoskeletal pain. The findings from the literature review, developmental studies and stakeholder meetings were used to inform decisions regarding (i) the target group of the intervention(patients); (ii) the underlying model driving the content of the intervention; (iii) the mode of delivery of the intervention; (iv) who was to deliver the intervention; (v) the structure, content and methods of delivery of the training and mentoring package.

## Results

### The Target Group of the Intervention

The target group of the intervention was identified as a result of the developmental work, it consisted of working age adult primary care consulters who were employed (paid) and who were currently absent for < 6 months duration (either GP or self-certified absence) due to musculoskeletal pain, or who were assessed by their GP or nurse practitioner as struggling with work due to musculoskeletal pain. The results of the literature review highlighted the costs to individuals and society of sickness absence and struggling at work. Although the literature supports early intervention, at the time the SWAP trial was designed existing services in the UK were largely restricted to those absent from work for > 6 months (e.g., via Jobcenter Plus). This was also an issue highlighted by the consultations with clinicians, with many concerned that patients needed to spend 6 months away from the workplace before they were able to access any occupational health support through national schemes. This concern was compounded when consideration was given to people employed in Small and Medium Enterprises (SMEs), with just 34% of SME employees having access to occupational health services [34], which leaves a significant proportion of people without adequate support. In order to address this, the new ‘early’ intervention designed as part of the SWAP trial needed to be available to people who were likely to fall into this gap in service, i.e. those who were absent from work for < 6 months, and were either self-employed or employed at any type of organisation.

### The Content of the Vocational Advice Intervention

From the literature review it was clear that people who fail to enter, stay at, or return to work don’t generally have a more serious health condition or a more serious injury than those who return to work [22]. They tend to have common health problems. In principle those problems should be manageable, but health, personal, psychological, and social/occupational obstacles can severely impede recovery and participation [22]. Thus the VAI needed to take into consideration occupational, psychosocial and health related obstacles to work. The Psychosocial Flags model of management of the heath and work interface was developed to aid the understanding and identification of obstacles to work participation [22]. A practical framework developed from the Psychosocial Flags model [22] recommends that a biopsychosocial health and work assessment is conducted to identify an individual’s obstacles to staying at work (SAW) or RTW. This should focus in particular on three categories of obstacles to SAW/RTW: Yellow flags: individual patients’ thoughts, feelings and behaviours about their health and personal capabilities in dealing with symptoms; Blue flags: perceptions of work and its potential impact on symptoms and work capability; Black flags; aspects of the working conditions and wider social-economic context (principally concerning factors outwith the person’s sphere of influence) [22]. Once obstacles to work have been identified, they should be addressed by developing a plan to overcome those obstacles—combining optimum clinical management, vocational rehabilitation and organisational interventions [10] and by following biopsychosocial principles—i.e. by including elements which aim to increase activity levels and restore function (biological), change behaviour, shift perceptions, attitudes and beliefs in personal and work life (psychological), and provide support with the involvement of the employer where appropriate (social) [20]. The literature suggested that it is helpful to work with the employee and key stakeholders to problem solve and develop an individualised written plan to overcome any health and work related obstacles [22]. This should clearly describe the level, type and frequency of any interventions, including the next steps, persons’ responsible and agreed timelines. In order to facilitate a timely, safe and sustained RTW a written RTW plan can be negotiated. This could involve transitional work arrangements (temporary modifications to the job), such as a gradual return to the original job using staged increases in hours and days worked, a return to partial duties of the original job, modifications to the workplace or work equipment, or temporary/permanent redeployment to another job, as appropriate [22]. Throughout the process, the employee should be central to the SAW/RTW process because a successful outcome is unlikely without their ownership of their work and health situation and their commitment to the plan [35]. If there are health related obstacles to SAW/RTW [20], the plan might include facilitating timely access to new or existing work-focused health care services. More detail about potential obstacles to SAW/RTW and what the VAIs were to do to overcome them can be found in Table 1. As part of the VAI, the patient’s situation and progress was regularly re-evaluated until SAW/RTW was achieved or until they were discharged. Patients were discharged by the VAs if:

1. The patient did not wish to continue
2. The obstacles to SAW/RTW were not modifiable by the VAs (e.g. bullying or harassment issues, litigation) in which case the patient was signposted to more appropriate support services
3. A sustained RTW (defined as maintenance of work for 1 month) had been achieved
4. The patient felt able to manage their health condition in the context of their work
5. The patient had been absent from the workplace for a total of 6 months, at which point they were directed towards other appropriate services (e.g. Jobcentre Plus)

**Table 1:** Common obstacles to SAW/RTW and the actions the VAs were to take to overcome them

Flag	Description	Obstacles	Potential actions
Yellow	Primarily psychological in nature and clinical in focus and encompass people’s beliefs about their health problem	• Unhelpful/erroneous beliefs and expectations about their health • Poor expectations of recovery • Preoccupation with health • Worry/distress/anxiety/depression • Fear of movement (kinesiophobia) • Passive coping strategies	• Provide reassurance and a rational explanation • Set realistic expectations • Dispel myths • Provide advice and support to return to or remain active • Facilitate referral to health care providers and liaise to ensure health care interventions are timely, appropriate and rehabilitation/work focused • Build self-efficacy • Refer back to the GP if further clinical support was needed e.g. mental health assessments • Liaise and maintain communication with employers • Suggest suitable adjustments at work
Blue	Refer primarily to *perceptions* of work (e.g. relationships with managers, control over workload)	• Unhelpful beliefs about the relationship between work and health (e.g. the belief that one has to be pain free before returning to work, or that it is not possible to do a manual job, with low back pain) • Fear of re-injury • Physically demanding job • High job demands • Low expectations of resuming work • Low job satisfaction • Low social support	• Build the importance/value of work • Convey positive but realistic messages about participant’s ability to work • Gently challenge unhelpful/erroneous beliefs about health and work and their inter-relationship • Provide evidence-based advice and information • Encourage patients to remain or return to activity, including work related movements/activities and to RTW, as soon as is possible • Provide the GP with accurate information about the patient in order to inform the process of issuing a Fit Note (https://www.gov.uk/government/collections/fit-note), if required • Refer to workplace occupational health (if available) • Facilitate the patient to develop a RTW plan in collaboration with key stakeholders • Identify a person in the workplace the patient could talk to i.e. line manager • Agree a return to work plan/implement graded return to work • Maintain regular contact with workplace • Encourage attendance at work meetings/social events • Use transitional work arrangements i.e. modified duties
Black	Refer to more *objective* features of workplace, such as working conditions and sickness absence entitlements/policies	• Misunderstanding/disagreements with employers • Financial and/or compensation problems • Lack of family support/inadequate support • Social isolation/dysfunction • Unhelpful company policies or procedures • Broader determinants of poor health and work impairment such as housing or financial concerns (e.g. wage replacement benefits, debts)	• Identify key stakeholders (employee, GP, other health care providers, employers and any other stakeholders) and their role in SAW/RTW facilitation • Take into account different stakeholders views, provide coordination, facilitate communication and co-operation in order to overcome modifiable obstacles and to ensure a goal-oriented approach to achieving specific work retention and an early and sustainable RTW • Empower the patient to liaise with the workplace or liaise directly with the workplace, in an open and transparent way, with the purpose of identifying issues, agreeing the RTW plan and addressing modifiable obstacles • Arrange a worksite visit and meeting, if necessary • Use a problem solving approach to tackle obstacles • Emphasise ability not disability and manage expectations around symptoms and work • Encourage the workplace to consider adaptations or changes to the patient’s work situation, transitional work arrangements (phased return to specific restricted duties, flexible working, access) or a change in job role and responsibilities (DH/DWP, 2008) if indicated • Ensure a timely start to the RTW process • Signpost to information/services for help with wage replacement benefits, debt, housing, legal and other issues

This table is adapted from ‘Tackling Musculoskeletal Problems: A Guide for the Clinic and Workplace—Identifying Obstacles Using the Psychosocial Flags Framework’ [22]

### The Mode of Delivery of the Vocational Advice Intervention

Vocational case management involves providing coordination, facilitating communication, and working collaboratively with treatment providers, the employee, the workplace and any other key stakeholders, to ensure a goal-oriented approach to achieving work retention and an early and sustainable return to work, and has been shown to be an effective RTW intervention, which can reduce healthcare costs, treatment duration, time off work, and improve worker productivity [36]. The Psychosocial Flags Framework incorporates the principles of stepped care, which is a suitable model of healthcare delivery when people cannot reliably be stratified to the most appropriate level of care in the first instance. In stepped care the interventions are sequenced so that the intensity, complexity, and costs of care are guided by each patient’s observed outcome [37]: patients access the lowest (least intensive, complex, costly and burdensome) step first and only progress (step up) onto the next step if they do not benefit from simpler first-line intervention, show inadequate response or can be accurately predicted not to benefit [22]. Stepped care enables patients’ progress to be regularly reviewed and acted upon to allow the intervention to be stepped up or stepped down [10]. In the SWAP study the steps consisted of: step one: initial phone call; step two: subsequent phone calls and/or a face to face appointment; step three: further face to face appointments (including an optional worksite visit), with the VAs commencing with the initial phone call and only “stepping up” support for participants when necessary. Because the VAI in the SWAP study was an early intervention, the research team anticipated that many patients would only require step one, an initial telephone call to develop and agree the SAW/RTW plan. There is evidence to suggest that telephone based vocational advice, when delivered by appropriately trained professionals, can help a substantial proportion of people to self-manage their health problem and may also facilitate RTW [38]. Further details of the steps are reported in Table 2.

**Table 2:** The steps in the VAI

Step one	Step two	Step three
The initial telephone call: All patients referred to the service were to receive a telephone call from a VA. It was envisaged that evidence-based advice, information and support might be all that was required for some patients and that they would successfully SAW/RTW after one or more telephone calls. The VA’s were to identify patients who required further support to address modifiable obstacles to SAW/RTW (e.g. if patients were not confident to SAW/RTW and/or couldn’t specify when they would RTW), in which case, arrangements were made to “step-up” to a face-to-face meeting in order to provide these patients with additional support	Subsequent phone calls and/or a face to face appointment: Following the initial phone call, further phone calls and/or a face to face appointment were to be scheduled, as appropriate. The face-to-face appointment(s) were to take place at the GP practice, at a mutually convenient day and time. The duration of these appointments was to be flexible, depending on what was required	Further face-to-face appointments (including an optional worksite visit): If more than one face-to-face appointment was indicated then these were to be arranged. In addition, the VAs were to conduct a worksite visit/meeting if required, in order to identify issues, problem solves difficulties, agree and implement an action plan and agree the RTW goal

### Determining Who Should Deliver the Vocational Advice Intervention

Communication between different stakeholders (e.g. the worker, employer, HCPs) and coordination in the RTW process can be challenging [39]. It has therefore been argued that a HCP could act as an intermediary, by facilitating communication, by gaining a good understanding of the employee’s needs, liaising between other HCPs and the employer and by visiting the workplace [39]. A review and stakeholder consultation of early interventions in primary care to prevent long-term incapacity for work suggested that vocational support can be delivered by a multidisciplinary team or by a single HCP [35]. However, it is considered important that the vocational case manager is not the caring clinician, because HCPs with a clinical role can revert to delivering health care, rather than focusing on vocational case management [36]. Some studies have used a combination of HCPs from different professional backgrounds or a combination of HCPs and non-HCPs [36], but it seems the skill base of successful vocational case managers is more important than their professional training or background [36]. It was felt that HCPs offering the VAI needed to become integrated within general practice, be able to navigate the health care system and have credibility with health care providers, patients and employers when giving information and advice about managing health conditions in the workplace. The number and types of professionals involved in providing vocational advice in the UK are limited compared to those in the US and many other countries. Most people in the UK do not have access to vocational rehabilitation or occupational health through their workplace, and as such their GP and primary care team are their main source of advice about work [34]. The intention in the SWAP trial was therefore to embed the intervention into existing primary care, thus we wished to find out if we could successfully train the range of personnel available in this environment, [GPs, Allied Health Professionals (AHPs) and nurses], at a reasonable cost. Findings from the literature review and meetings with clinicians suggested that GPs did not feel able to take on the role of delivering the VAI, given the limited duration of GP consultations and the demands on their time. It was felt instead, that AHPs and nurses would be ideally placed to take on the VA role and earlier developmental work suggested that physiotherapists may be well placed to adopt this role as they already ask about work [32]. The role was therefore advertised to nurses and AHPs. Four healthcare practitioners were recruited to be trained to deliver the VAI; three physiotherapists and one nurse (all vocational advice was actually delivered by the three physiotherapists).

### The Location of the VA Service

There is support for locating a vocational intervention within a Department for Work and Pensions (DWP) facility [40]. However, there is an argument for the delivery of vocational advice to be seen as clearly independent of the fit note and benefits system, a view that was strongly supported by PPIE members during the stakeholder meetings. The location of a vocational intervention also impacts on the timing of the intervention, primary care enables early access whereas services sited elsewhere tend to result in late contact. Whilst primary care and occupational health may both be appropriate settings in which to identify people struggling at work, most people in the UK do not have access to occupational health through their work, their GP and primary care team are therefore their main source of advice about work [19, 41]. In the SWAP study the VAs were therefore embedded within the GP practices in order to intervene early, develop close working relationships and ensure good communication with primary HCPs.

### The Structure, Content and Methods of Delivery of the Training and Mentoring Package

The literature suggests that GPs and physiotherapists lack adequate training or expertise in how to assess or manage work related issues [30] and their clinical practice is not always in keeping with evidence based guidelines when it comes to advice about work and health [42]. In order to ensure the VAs were able to deliver evidence-based vocational advice and support based on the Psychosocial Flags Framework [22], stepped care, and case management, the research team developed a bespoke training package, built on the VAs’ existing expertise as HCPs, in order to equip them with the necessary skills to identify obstacles to SAW/RTW and support patients by collaboratively developing and implementing a plan to overcome them. The training was designed to take into account the biopsychosocial nature of the intervention, the VAs’ professional background, the study population, and the UK context (e.g. social security system, and employment law and practice). The training addressed a number of learning objectives relating to the key attitudes, knowledge, skills and behaviours that were deemed essential for effective delivery of the VAI. See Table 3 for further information about the knowledge and skills included on the training course.

**Table 3:** The key knowledge and skills included on the training course

Knowledge	Skills
• Epidemiology of presenteeism and sickness absence • The value of work to health • Evidence concerning the relationship between work and heath and what works in vocational rehabilitation and vocational case management • Vocational case management and how it differs from the clinical role and from clinical case management • The Flags model of health and work • Obstacles to SAW/RTW • Stepped care • The hierarchy of RTW goals • How to overcome obstacles to RTW • Key internal and external stakeholders (the likely drivers of their behaviour, their possible concerns and roles) and considerations when communicating with stakeholders (when, why, who and how) • Best practice in developing a RTW plan • Best practice in organising and conducting a worksite visit/meeting • Relevant sickness absence and employment related legislation, policy and practice • UK work and social security system context • Resources-written information, online resources and other services • When and how to discharge	• Communication skills and motivational interviewing • How to explain the VA role and service • Obtaining consent • Eliciting relevant information in order to ascertain the patients work and health situation and any obstacles to SAW/RTW • Making sense of assessment finding and identifying obstacles to SAW/RTW • Correcting unhelpful beliefs about the value of work to health or the relationship between work and health (e.g., through the provision of verbal and/or written evidence based information) • Conducting the telephone consultation and face to face meetings and completing the computer and paper based documentation • Responding to frequently asked questions or concerns • Collaborative goal setting • Case management • Problem solving RTW—action planning, monitoring and modifying plans • Developing a written RTW plan • Providing information and signposting patients to additional sources of information or assistance • Setting up, conducting and following up after a work site visit/meeting

The methods of training delivery were based on a previously developed approach to training AHPs in good management of musculoskeletal pain [43]. They included self-directed study, Powerpoint presentations, case based discussion, paper based activities, role-play, demonstration and computer based activities. In terms of the duration of training, Hanson et al. [36], for instance, delivered 14 days training to vocational case managers. However, it was neither feasible nor necessary for the VAs in this study to have this intensity of training because of their existing skills and understanding, the nature of the VAI (early and largely telephone based), and the less complex needs of this study population (at work but struggling or very early in their sickness absence). The training in SWAP therefore consisted of a face-to-face course delivered over 4 days, 1 day/week for 4 weeks, with a refresher training day 3 months later. Training was provided by an experienced Consultant Physiotherapist (GS) with clinical, teaching, research and service development expertise in vocational rehabilitation and in developing and delivering training and mentoring packages as part of clinical trials [43]. The VAs were given written resources to support them in developing their knowledge and skills and to use with their patients. Studies have highlighted the value of ongoing support (e.g. mentoring) after initial training programmes are complete [44]. In light of this, mentoring for the VAs in the SWAP study was provided to help ensure the VAs put into practice what they had learnt (fidelity) and continued to do so over the longer term (sustainability). Mentoring served to consolidate and further develop the VAs knowledge, skills and confidence and to support them to “embed” the skills learnt in training into clinical practice. The VAs received monthly mentoring sessions as a group lasting 1.5–2 h throughout the 12 month duration of the trial. The mentoring was delivered by the trainer who provided the VA training course (GS) and also by a clinical/health psychologist (CM) with experience of the clinical/occupational interface in the context of musculoskeletal management, and expertise in training health-care professionals in psychologically informed practice [27, 45]. A variety of methods were used iteratively to maximize the effectiveness of mentoring (e.g. role play; discussion, sharing of information and resources), discussion focused on the VAs’ patient cases, implementation of the VAI and any themes that the VAs were struggling with.

## Discussion and Conclusions

There were both strengths and limitations to the method of intervention and training development. Whilst we did not use formal methods such as intervention mapping, the process of intervention and training development was consistent with the new Medical Research Council guidance on developing and evaluating complex interventions [18]. Evidence was drawn from developmental studies; literature and policy documents; consultation with health professionals and patients, but a formal systematic review was not conducted and at times it was difficult to draw firm conclusions because evidence in some areas (early intervention, delivered by primary care clinicians) was limited. The development process led to a number of conclusions: the VAI needed to be implemented as early as was feasible (before 6 months absence from work); people struggling at work face the same sort of obstacles to SAW as those on short-term absence do for return to work; the intervention needed to be accessible to those who are “at risk” of work absence due to their pain condition as well as those who are currently sick-listed; the intervention needed to be clearly independent of the fit note and benefits systems; the intervention should be staffed by individuals with a knowledge of NHS healthcare; the VAI should adopt the Psychosocial Flags Framework approach to management of the heath and work interface; incorporate the principles of stepped care along with a case management approach; be primarily delivered through the telephone; involve work accommodations; the training package should aim to equip the VAs to deliver the VAI by paying specific and systematic attention to identifying and overcoming modifiable obstacles to SAW/RTW; and regular mentoring meetings should be scheduled to ensure that VAs are supported to deliver the VAI consistently. A VAI was subsequently developed which incorporated all of these elements, along with a training and mentoring package to support the VAs to deliver it. Previous examples of integrated health and occupational support and early interventions in Sweden and the Netherlands have led to fewer days work absence, earlier return to work and reductions in health care use [46, 47], but many studies focus on the workplace only and not the interface between health and work, few have tested interventions to manage work absence in those with health conditions [47, 48] and few have been conducted in the UK. Of those studies that have tested interventions to manage work absence in those with health conditions [47, 48], the Fit for Work Service pilots tested different models of delivering vocational advice however only 21% of referrals came from general practice [48]. A more recent UK study aiming to provide vocational advice in primary care lacked GP engagement leading to poor recruitment [49]. The SWAP trial was therefore the first to provide an early VAI, embedded in UK general practice, to people with musculoskeletal pain, a leading cause of work absence. The results of the trials showed that the VAI significantly reduced days absent over 4 months and was cost effective compared to best care [16], however, exploratory subgroup analysis in those participants with < 10 days absence versus ≥ 10 days but < 6 months absent at baseline found that the intervention was more successful in those with the longer absence duration. These results suggest that a VAI might be better targeted to those with > 10 days (2 working weeks) of absence. The VAI was developed in the context of UK primary care, however, the evidence underpinning the VAI was drawn from an international perspective and the key components of the VAI (case management, stepped care, Flags model) therefore have the potential to be applied to any country where there is access to primary care or occupational healthcare. Whilst this intervention has not yet been tested in other settings or countries the publication of the development and content of the VAI means that it can more easily be tested for generalisability. Full results of the SWAP randomised trial, which tested the VAI, have recently been published [16].
